# Volatiles in Inter-Specific Bacterial Interactions

**DOI:** 10.3389/fmicb.2015.01412

**Published:** 2015-12-18

**Authors:** Olaf Tyc, Hans Zweers, Wietse de Boer, Paolina Garbeva

**Affiliations:** ^1^Department of Microbial Ecology, Netherlands Institute of EcologyWageningen, Netherlands; ^2^Department of Soil Quality, Wageningen University and Research CentreWageningen, Netherlands

**Keywords:** volatolomics, soil bacteria, *Chryseobacterium*, *Dyella*, *Janthinobacterium*, *Tsukamurella*, inter-specific interactions, volatile activities

## Abstract

The importance of volatile organic compounds for functioning of microbes is receiving increased research attention. However, to date very little is known on how inter-specific bacterial interactions effect volatiles production as most studies have been focused on volatiles produced by monocultures of well-described bacterial genera. In this study we aimed to understand how inter-specific bacterial interactions affect the composition, production and activity of volatiles. Four phylogenetically different bacterial species namely: *Chryseobacterium, Dyella, Janthinobacterium*, and *Tsukamurella* were selected. Earlier results had shown that pairwise combinations of these bacteria induced antimicrobial activity in agar media whereas this was not the case for monocultures. In the current study, we examined if these observations were also reflected by the production of antimicrobial volatiles. Thus, the identity and antimicrobial activity of volatiles produced by the bacteria were determined in monoculture as well in pairwise combinations. Antimicrobial activity of the volatiles was assessed against fungal, oomycetal, and bacterial model organisms. Our results revealed that inter-specific bacterial interactions affected volatiles blend composition. Fungi and oomycetes showed high sensitivity to bacterial volatiles whereas the effect of volatiles on bacteria varied between no effects, growth inhibition to growth promotion depending on the volatile blend composition. In total 35 volatile compounds were detected most of which were sulfur-containing compounds. Two commonly produced sulfur-containing volatile compounds (dimethyl disulfide and dimethyl trisulfide) were tested for their effect on three target bacteria. Here, we display the importance of inter-specific interactions on bacterial volatiles production and their antimicrobial activities.

## Introduction

Soil bacteria produce an astounding array of secondary metabolites. Gaseous secondary metabolites, commonly known as volatile organic compounds (VOCs) are small molecules (<300 Da) belonging to different chemical classes that can evaporate and diffuse easily through air- and water-filled pores (Schulz and Dickschat, 2007; Penuelas et al., 2014). These physiochemical properties make volatiles ideal metabolites for communication and antagonistic interactions between soil microorganisms living at a certain distance from each other. Indeed, recent studies indicate that soil microorganisms can employ volatile compounds as info-chemicals, growth stimulants, growth inhibitors, and inhibitors of quorum-sensing (Kai et al., 2009; Chernin et al., 2011; Effmert et al., 2012; Kim et al., 2013). Furthermore, rhizosphere bacteria emit volatiles that can promote plant growth and elicit induced systemic resistance (ISR) and induced systemic tolerance (IST) in plants (Ryu et al., 2003, 2004). However, the role of volatiles in competitive interactions between soil bacteria is so far poorly understood.

In the past few years the research on volatiles emitted by bacteria received increased attention from a more applied point of view as these compounds have intriguing properties which are of great interest for agriculture (pathogen suppression), food preparation (aroma), and cosmetics industry (perfume odors; Krings and Berger, 1998; Wheatley, 2002; Beshkova et al., 2003; Schwab et al., 2008; Deetae et al., 2009; Effmert et al., 2012; Kanchiswamy et al., 2015).

Bacterial volatiles belong to different chemical classes like alkenes, alcohols, ketones, terpenes, benzenoids, pyrazines, acids, and esters. However, the composition of emitted volatiles (volatile blend composition) may vary with cultivation conditions, in particular with respect to the substrate composition of the growth media (Cleason, 2006; Blom et al., 2011; Groenhagen et al., 2013; Garbeva et al., 2014a). Other factors known to influence volatile production are microbial physiological state, oxygen availability, moisture, temperature and pH (Bjurman, 1999; Insam and Seewald, 2010; Romoli et al., 2014).

The technical developments that have been made in recent years in the field of mass spectrometry have led to the improvement of volatile compounds detection. The details of these developments have recently been summarized by Carter (2014). However, the main challenge in volatolomics is the ability to identify and quantify the entire set of emitted volatiles. The detected volatile blends are mostly quite complex and make the identification of biologically relevant volatiles a demanding and challenging task (Farag et al., 2012; Tait et al., 2014).

To date more than over 1000 microbial volatiles are reported and described in a special database for microbial VOCs called mVOC^1^ (Lemfack et al., 2014). Nevertheless, this number is rather low compared to the high diversity of bacterial taxa in soil, suggesting a big underestimation of the actual real number of microbial volatiles (Kai et al., 2009; Lemfack et al., 2014). Moreover, most of the studies on microbial volatile detection have dealt with monocultures of already well-described bacterial genera. Thus, very little is known on how inter-specific interactions affect the volatile production. The investigation of volatiles production in more complex communities is of great interest since it could help to reveal the ecological role of these compounds. In the last years several independent studies reported that the production of secondary metabolites by soil bacteria can be influenced by interactions with microorganisms in their vicinity (Garbeva et al., 2011b; Traxler et al., 2013; Tyc et al., 2014). A high-throughput screening performed recently in our lab revealed that interactions between soil bacterial species have major effects in both directions: induction and suppression of antimicrobial activity (Tyc et al., 2014).

In this study we aimed to understand how inter-specific bacterial interactions affect the emission of volatiles and their activity. For this we selected four strains belonging to different bacteria species that have been isolated from the soil bacterial community associated with sand sedge (*Carex arenaria* L.) namely *Chryseobacterium* sp. AD48, *Dyella* sp. AD56, *Janthinobacterium* sp. AD80, and *Tsukamurella* sp. AD106 (Tyc et al., 2014). In an earlier screening it was observed that these bacteria showed induced antimicrobial activity during interactions but not in monocultures. In the current study, it was examined if these observations were also reflected by the volatiles emission. To this end the effects of volatiles on growth of fungal, oomycetal, and bacterial model organisms produced by the bacteria in monocultures as well in pairwise combinations were tested. Our overall hypothesis is that the blend composition volatiles produced during interactions differs from that of monocultures and consequently has different effect on model target organisms.

## Materials and Methods

### Bacteria and Culture Conditions

The bacterial isolates applied in this work were selected based on a previous observations of antimicrobial activity triggered by inter-specific interactions (Tyc et al., 2014). Four bacterial isolates were used: *Chryseobacterium* sp. AD48 (Class: *Flavobacteriia*) GenBank: KJ685263, *Dyella* sp. AD56 (Class: *Gammaproteobacteria*) GenBank: KJ685269, *Janthinobacterium* sp. AD80 (Class: *Betaproteobacteria*) GenBank: KJ685292, and *Tsukamurella* sp. AD106 (Class: *Actinobacteria*) GenBank: KJ685317. The bacterial isolates were pre-cultured from -80°C glycerol stocks on 1/10th TSBA (5.0 g L^-1^ NaCl, 1.0 g L^-1^ KH_2_PO_4_; 3 g L^-1^ oxoid tryptic soy broth (TSBA); 20 g L^-1^ Merck Agar, pH 6.5; Garbeva and de Boer, 2009) and incubated for 3 days at 24°C before starting the experiments.

To test the effect of bacterial volatile compounds on bacterial growth and colony morphology three indicator bacteria were used: *Escherichia coli* WA321, *Staphylococcus aureus* 533R4 (Meyer and Schleifer, 1978; Tyc et al., 2014) and *Serratia marcescens* P87 (Garbeva et al., 2014b). All three indicator bacteria were pre-cultured from -80°C glycerol stocks either on LBA media (LB-Medium Lennox, Carl Roth GmbH + Co. KG, Netherlands, art.no. X964.2, 20 g L^-1^ Merck Agar; *E. coli* WA321 and *S. aureus* 533R4; Sambrook and Russell, 2001) or on 1/10th TSBA (*S. marcescens* P87). The indicator organisms *E. coli* and *S. aureus* were incubated overnight at 37°C prior application, *S. marcescens* P87 was incubated at 24°C for 4 days prior usage. All bacterial isolates used in this study are listed in **Table 1**.

**Table 1:** Bacterial, fungal, and oomycetal organisms used in this study.

Strain	Phylum/class	GenBank	Reference	Function
**Volatile producing bacteria tested**				
*Chryseobacterium* sp. AD48	Flavobacteriia	KJ685263	Tyc et al., 2014	Used for volatile analysis
*Dyella* sp. AD56	Y-proteobacteria	KJ685269	Tyc et al., 2014	
*Janthinobacterium* sp. AD80	β-proteobacteria	KJ685292	Tyc et al., 2014	
*Tsukamurella* sp. AD106	Actinobacteria	KJ685317	Tyc et al., 2014	
**Fungal/oomycetal test organisms**				
*Rhizoctonia solani* AG2.2IIIB	Basidiomycota	KT124637	Garbeva et al., 2011b	Eukaryotic model organisms for growth inhibition
*Pythium ultimum* P17	Oomycete	KT124638	Garbeva et al., 2014b	
*Fusarium culmorum* PV	Ascomycota	-	Garbeva et al., 2014b	
**Bacterial test organisms**				
*Serratia marcescens* P87	Y-proteobacteria	-	Garbeva et al., 2014b	Bacterial model organisms for growth inhibition and colony morphology changes
*Escherichia coli* WA321 DSMZ 4509	Y-proteobacteria	-	Tyc et al., 2014	
*Staphylococcus aureus* 533R4 Serovar 3 DSMZ 20231	Firmicutes	LN681573	Meyer and Schleifer, 1978	

### Cultures and Growth Conditions of Fungi and Oomycetes

The fungi *Rhizoctonia solani* AG2.2IIIB and *Fusarium culmorum* PV and the oomycete *Pythium ultimum* P17 were used in this study (Garbeva et al., 2014b). The fungi and oomycete were pre-cultured on 1/5th potato dextrose agar (PDA; 29 g L^-1^ Oxoid CM 139; Fiddaman and Rossall, 1993) and incubated at 24°C for 7 days prior usage. All fungal and oomycetal organisms are listed in **Table 1**.

### Experimental Treatments

Ten different treatments were performed in triplicates. These treatments were: monoculture 1 (*Chryseobacterium* sp. AD48), monoculture 2 (*Tsukamurella* sp. AD106), monoculture 3 (*Dyella* sp. AD56), monoculture 4 (*Janthinobacterium* sp. AD80) and pairwise interaction of the isolates: interaction 1 (*Chryseobacterium* sp. AD48 + *Tsukamurella* AD106), interaction 2 (*Dyella* sp. AD56 + *Janthinobacterium* sp. AD80), Control 1 (glass Petri dish with TSBA media without inoculated bacteria, as background control in GC/MS measurement), Control 2 (two compartment Petri dish inoculated with model organisms without exposure to bacterial volatiles), Control 3 (top bottom Petri dish inoculated with fungal/oomycetal model organisms without exposure to bacterial volatile compounds). Control 4 (two compartment Petri dish inoculated with model organisms without exposure to the tested pure volatile compounds). The effect of the produced volatiles was tested on fungal, oomycetal, and bacterial growth via determination of hyphal biomass or growth inhibition assays. For the inoculation of the experiments a single colony of each test isolate was picked from a plate and inoculated in 20 mL 1/10th TSB (5.0 g L^-1^ NaCl, 1.0 g L^-1^ KH_2_PO_4_; 3 g L^-1^ TSBA) and incubated overnight at 24°C, 220 rpm. On the next day the OD_600_ of each isolate was measured on a GENESYS^TM^ 20 spectrophotometer (Thermoscientific, Netherlands, Cat# 4001-000) and a inoculation suspension for each treatment was prepared in 20 mL of 10 mM P-Buffer (pH 6.5) containing bacterial cells in a concentration of ∼1 × 10^∧^5 CFU/mL.

### Volatile Trapping

Next to the inhibition experiments, bacterial volatiles emitted in monocultures and pairwise combinations were trapped and analyzed. For trapping of VOCs emitted by bacteria a volume of 100 μl of inoculation suspension was spread on 1/10th TSBA (20 mL) in glass Petri dishes designed for headspace volatile trapping (Garbeva et al., 2014b). The Petri dishes were closed by a lid with an outlet connected to a steel trap containing 150 mg Tenax TA and 150 mg Carbopack B (Markes International, Ltd., Llantrisant, UK; Supplementary Figure S1). All treatments were inoculated in triplicate. The volatiles were collected after 48 and 72 h of incubation and the Tenax steel traps were stored at 4°C until GC-Q-TOF analysis.

### GC-Q-TOF Analysis

The trapped VOCs were desorbed from the traps using an automated thermodesorption unit (Unity TD-100, Markes International, Ltd., Llantrisant, UK) at 210°C for 12 min (He flow 50 mL/min) and trapped on a cold trap at -10°C. The trapped volatiles were introduced into the GC-QTOF (model Agilent 7890B GC and the Agilent 7200A QTOF, Santa Clara, CA, USA) by heating the cold trap for 3 min to 280°C. Split ratio was set to 1:10, and the column used was a 30 mm × 0.25 mm ID RXI-5MS, film thickness 0.25 μm (Restek 13424-6850, Bellefonte, PA, USA). Temperature program used was as follows: 39°C for 2 min, from 39 to 95°C at 3.5°C/min, then to 165°C at 6°C/min, to 250°C at 15°C/min and finally to 300°C at 40°C/min, hold 20 min. The VOCs were detected by the MS operating at 70 eV in EI mode. Mass spectra were acquired in full-scan-mode (30–400 AMU, 4 scans/s). Mass-spectra’s were extracted with MassHunter Qualitative Analysis Software V B.06.00 Build 6.0.633.0 (Agilent Technologies, Santa Clara, CA, USA) using the GC-Q-TOF qualitative analysis module. The obtained mass spectra’s were exported as mzData files for further processing in MZmine V2.14.2. The files were imported to MZmine V2.14.2 (Copyright 2005–2012 MZmine Development Team; Katajamaa et al., 2006; Pluskal et al., 2010) and compounds were identified via their mass spectra using deconvolution function (Local-Maximum algorithm) in combination with two mass-spectral-libraries: NIST 2014 V2.20 (National Institute of Standards and Technology, USA^2^) and Wiley 7th edition spectral libraries and by their linear retention indexes (LRIs). The LRI values were calculated using an alkane calibration mix before the measurements in combination with AMDIS 2.72 (National Institute of Standards and Technology, USA). The calculated LRI were compared with those found in the NIST and in the in-house NIOO LRI database. After deconvolution and mass identification peak lists containing the mass features of each treatment (MZ-value/Retention time and the peak intensity) were created and exported as CSV files for statistical processing. The whole volatolomic workflow is shown in Supplementary Figure S2.

### Bioassay for Testing the Effect of Bacterial Volatiles on Fungal and Oomycete Growth

To test the effect of the emitted bacterial volatiles on fungal/oomycete growth the hyphal extension and biomass were measured. The assays were performed in Petri dishes containing top and bottom growth areas (Supplementary Figure S3). At the bottom of the Petri dish, 100 μl of bacterial suspensions in 10 mM phosphate buffer (pH 6.5) containing ∼1 × 10^∧^5 CFU/mL were spread on 20 mL 1/10th TSBA. At the lid of the Petri dish 12.5 mL of water-agar medium (WA; 20 g L^-1^ MERCK agar) was added and inoculated in the middle with a 6-mm-diameter PDA agar plug containing fungal (*R. solani, F. culmorum*) or oomycete (*P. ultimum*) hyphae. The plates were sealed with two layers of parafilm and incubated at 24°C for 5 days. In this way the tested fungi were exposed (without direct physical contact) to the volatiles produced by the bacteria in the bottom compartment. On the fifth day the extension of the hyphae was measured in 4 evenly spaced directions and compared to the hyphae extension in the control plates (fungi exposed to 1/10th TSBA growth medium without bacteria).

### Determination of Fungal and Oomycetal Biomass

Fungal biomass was determined as described by Garbeva et al. (2014b). The whole growth area in the lids containing water agar and fungal hyphae was cut in ∼2 cm^2^ pieces and transferred to a glass beaker containing 100 mL of sterile demi-water (H_2_O). The agar was melted for ∼2.5 min in a microwave oven (temperature increased to about 100°C). The melted agar containing the hyphae was filtered over a tea strainer and the remaining hyphae were rinsed with about 150–200 mL of hot water (∼80°C). The hyphae were picked with tweezers from the tea strainer and transferred to a micro centrifuge tube and stored at -20°C until analysis. For determination of fungal/oomycete biomass the frozen hyphae were transferred to a glass tube with lids with small holes and subjected to freeze-drying for 48 h (Labconco Freezone 12 with Labconco Clear Drying Chamber nr.7867000). The samples were stored in an exsiccator with dried silica gel for 3 h (Silica Gel Orange, 2–5 mm, indicator, Roth, art.nr.P077.2) prior weighing the dry biomass.

### Bioassay for Testing the Effect of Bacterial Volatiles on Growth and Colony Morphology of Target Bacteria

The assays were performed in two-compartment Petri dishes (Greiner bio-one B.V., Alphen a/d Rijn, The Netherlands, Cat# 635102) containing two separated compartments (Supplementary Figure S4). In such way the growth response of target bacteria to volatile producing bacteria could be determined without direct physical contacts. One compartment was supplemented with 12.5 mL TSBA and contained the volatile producing bacteria either in monoculture or in pairwise interactions. The second compartment contained the indicator bacteria and was supplemented either with 12.5 mL LBA (*E. coli* WA321, *S. aureus* 533R4) or with 12.5 mL TSBA (*S. marcescens* P87). The compartment for the volatile producing bacteria was inoculated with 100 μl bacterial suspensions master mix of monocultures or pairwise interactions prepared with 20 mL of 10 mM phosphate buffer (pH 6.5) containing ∼1 × 10^∧^5 CFU/mL. The compartment for the indicator organisms was inoculated with four droplets (5 μL) of each indicator bacteria. The droplets of the indicator bacteria were placed in a distance of 2 cm to each other and contained 1 × 10^∧^5, 1 × 10^∧^4, 1 × 10^∧^3, and 1 × 10^∧^2 CFU/mL of either *E. coli* WA321, *S. aureus* 533R4, or *S. marcescens* P87 (Supplementary Figure S4). As controls the first compartment of the Petri dish was kept empty. After 4 days of incubation at 24°C the plates were examined and digital photographs were taken. The digital images were analyzed using the AXIO VISION v4.8 imaging Software (Carl Zeiss Imaging Solutions GmbH, Germany) for enumeration and surface-area determination (in pixelˆ2) of the bacterial colonies. All treatments were performed in triplicate.

### Test of Pure Volatile Compounds on Bacterial Growth and Colony Morphology

The effect on growth, colony morphology and pigmentation by pure dimethyl disulfide (DMDS; CH_3_S_2_CH_3_), dimethyl trisulfide (DMTS; CH_3_S_3_CH_3_) and the mixture of both compounds was tested on *E. coli* WA321, *S. aureus* 533R4 and *S. marcescens* P87. The assays were performed in two-compartment Petri dishes (Greiner bio-one B.V., Alphen a/d Rijn, The Netherlands, Cat# 635102). Both compartments were supplemented with either 12.5 mL LBA (assay performed with *E. coli* WA321 and *S. aureus* 533R4) or with 12.5 mL TSBA (assay performed with *S. marcescens* P87). In one compartment a filter paper with a diameter of ∼5.5 mm (Whatman^TM^ filter paper Cat# 1003-150, 6 μm pore size) was placed on the agar surface in the middle of the compartment. Stock solutions with a concentration of 10, 1, and 0.1 μM of the pure volatile compounds (DMDS or DMTS) and the mixture of both compounds (DMDS + DMTS) were prepared by serial dilution of the pure compounds in Methanol (LiChrosolv^®^, Index-No: 603-001-00-X, Merck, Darmstadt, Germany). For the test a volume of 5 μl of each of the pure volatile stock solutions was added directly onto the filter paper resulting in a final concentration of 50, 5, and 0.5 μM, respectively. The other compartment was inoculated with the target bacteria *E. coli* WA321, *S. aureus* 533R4 or *S. marcescens* P87 by inoculating four spots in a distance of 2 cm from each other containing 1 × 10^∧^5, 1 × 10^∧^4, 1 × 10^∧^3, and 1 × 10^∧^2 CFU/mL (Supplementary Figure S4). As controls bacteria exposed to filter papers with no added volatile compounds were applied. The Petri dishes were sealed with a double layer of parafilm and incubated for 4 days at 24°C. After incubation digital photographs were taken and the effect on colony growth, colony morphology and pigment production (prodigiosin) in *S. marcescens* P87 was examined. All digital images were analyzed using the AXIO VISION v4.8 imaging Software (Carl Zeiss Imaging Solutions GmbH, Germany) for enumeration and surface-area determination (in pixelˆ2) of the bacterial colonies. All treatments were performed in triplicate.

### Statistical Analysis

Statistical analysis on volatolomic data was performed using the statistical analysis module of MetaboAnalyst V3.0, www.metaboanalyst.ca (Xia et al., 2012, 2015). Prior to statistical analysis data normalization was performed via log-transformation. To identify significant abundant mass features one-way-ANOVA with *post hoc* Tukey test (HSD- test) was performed between the data sets. To identify important mass features in the samples PLS-D analysis was performed. Mass features were considered to be statistical relevant if *p*-values were ≤0.05. Statistical relevant mass features were further used for the compound identification. Statistical analyses on fungal dry biomass and bacterial colony sizes were performed with IBM SPSS Statistics 23 (IBM, Somers, NY, USA) using one-way ANOVA and *post hoc* Tukey test between the data sets. The 5% level was taken as threshold for significance between control and volatile treatments.

### Determination of HCN, NH_3_ Emission, and pH Values in the Agar

All bacterial strains used in this study were tested for the emission of ammonia and HCN as well as for the ability to change the pH- value of the growth medium where the target organisms were inoculated. For these tests the bacteria were inoculated in two-compartment Petri dishes (start density ∼1 × 10^∧^5 CFU/mL) on 12.5 mL 1/10th TSBA. The second compartment was supplemented with 12.5 mL WA. After 4 days of growth the HCN and ammonia emission as well the pH-value of the target organism growth medium (WA) was determined. To test for the presence of Hydrocyanic acid the gaseous content of the Petri dish headspace was sucked through a Hydrocyanic acid test tube (Dräger Safety AG and CO. KGaA, Lübeck, Germany, order number: CH25701) using the Dräger accuro^®^ gas detection pump (Dräger Safety AG and CO. KGaA, Lübeck, Germany). Presence of Hydrocyanic acid was determined by color change of the test tube (formation of a red reaction product; Supplementary Figure S5). The pH of the target organism growth medium (WA) exposed to bacterial volatiles was determined by slightly pressing a pH test-strip VWR PROLABO dosatest^®^ (VWR international, Cat# 35309.606UK) for 30 s into the agar surface. The pH values were determined by color change of the test strip and compared to the color scale on the package (Supplementary Figure S6). The ammonia concentration was determined using the MQuant^TM^ ammonium test kit (Merck, Darmstadt, Germany, Cat# 110024) by placing a reaction activated test-strip on the lid of the Petri dish directly opposite to the bacterial culture and fixed with tape. The Petri-dish were closed and sealed with parafilm and incubated for 2 h at 24°C. After incubation the presence of ammonium was determined by color change of the test strip (Supplementary Figure S7).

## Results

### Detected Headspace Volatile Compounds and GC/MS-Q-TOF Analysis

**FIGURE 1:**
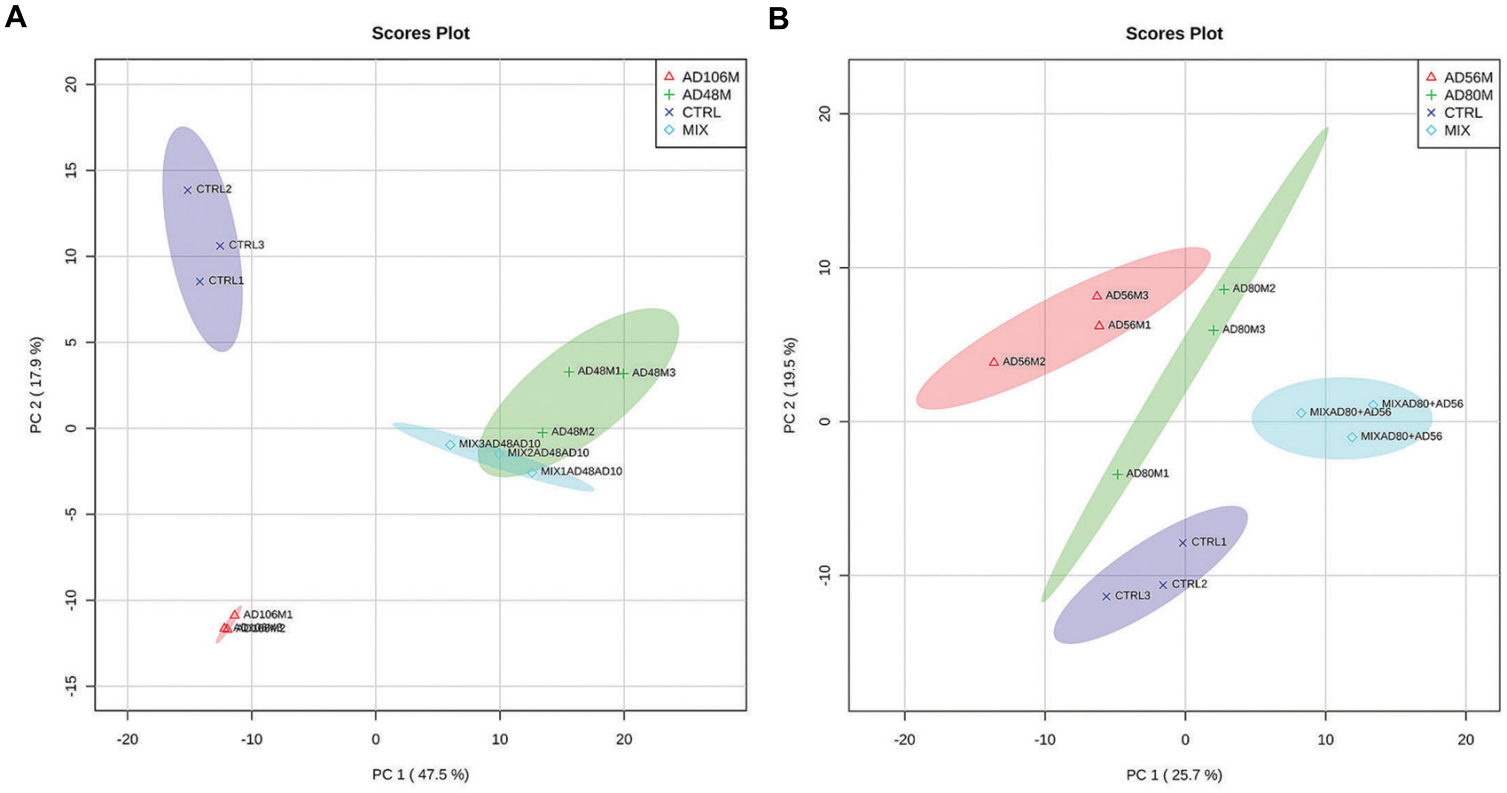
**PCA 2D- plots of volatiles emitted by monocultures and pairwise combinations of bacteria including confidence intervals (in semi-transparent colors). (A)** Monocultures and mixtures of *Tsukamurella* sp. AD106 and *Chryseobacterium* sp. AD48 and **(B)** monocultures and mixtures of *Dyella* sp. AD56 and *Janthinobacterium* sp. AD80.

GC/MS-Q-TOF based volatolomic analysis revealed a total number of 35 compounds that were not detected in the non-inoculated controls (**Table 2**). 27 compounds were obtained from the monocultures of *Chryseobacterium* sp. AD48, 15 compounds were obtained from the monocultures of *Tsukamurella* sp. AD106 and 26 compounds were detected in the interactions between these two bacteria (**Table 2**; **Figure 1A**). For the combinations of *Dyella* sp. AD56 and *Janthinobacterium* sp. AD80 we obtained a total number of 18 compounds, whereas 16 compounds were detected in the monoculture of *Janthinobacterium* sp. AD80 and only 13 compounds in the monoculture of *Dyella* sp. AD56 (**Table 2**; **Figure 1B**). We were able to tentatively identify 19 VOCs belonging to seven different chemical classes including alcohols, amines, esters, indole, thiocyanates, thioesters, and sulfides. However, a vast number of the detected compounds (*n* = 16) could not be assigned with certainty to a VOC and remained unknown. The most prominent detected headspace VOCs were sulfur containing compounds (such as sulfordioxide, methyl thioacetate, dimethyl sulfoxide, etc.). Two sulfur compounds DMDS (C_2_H_6_S_2_) and DMTS (C_2_H_6_S_3_) were produced by all bacteria (except DMTS which was not detected for *Janthinobacterium* sp. AD80).

**Table 2:** Tentatively identified volatile organic compounds emitted by four bacterial strains cultivated either in monoculture or in pairwise combination.

	Detected in treatment							
# Compound name/chemical class	RT^∗^	ERI^∗∗^	Chry	Tsuk	MIX Chry + Tsuk	Dye	Jant	MIX Jant + Dye
(1) Sulfurdioxide	2.58	521	x		x		x	x
(2) Cyclopentene	2.96	551	x		x	x	x	
(3) 2-Pentene	3.29	575				x		x
(4) Unknown compound 1	3.77	612	x	x	x	x	x	x
(5) Methyl isobutyrate	4.70	682				x		
(6) Methyl thioacetate	4.94	700	x		x		x	x
(7) Methyl thiocyanate	5.28	713		x			x	x
(8) 1-Butanol, 3-methyl-	5.69	728	x		x			
(9) Dimethyl disulfide	6.10	744	x	x	x	x	x	x
(10) Methyl isovalerate	6.86	769				x		
(11) *S*-methyl propanethioate	7.45	782	x		x		x	x
(12) 1,3 Dithiethane	7.64	786	x	x	x		x	x
(13) Dimethyl sulfoxide	8.46	806		x				
(14) 2,4-Dithiapentane	10.74	865	x	x	x		x	x
(15) Benzaldehyde	13.72	944	x	x	x		x	x
(16) Dimethyl trisulfide	14.33	960	x	x	x	x		x
(17) Unknown cycloalkane	16.86	1026	x	x	x	x	x	x
(18) Unknown branched alkene	17.39	1040	x	x	x	x	x	x
(19) Unknown sulfur containing compound	18.09	1058	x	x	x			
(20) 1,2,4-Trithiolane	19.30	1090	x	x	x		x	x
(21) Unknown compound 2	19.70	1101						
(22) Unknown compound 3	19.99	1110	x	x	x	x	x	x
(23) Unknown compound 4	20.63	1131	x		x			
(24) Dimethyl tetrasulfide	23.64	1227		x				
(25) Indole	25.82	1298	x					
(26) Butylhydroxytoluene	30.28	1540	x	x	x	x	x	x
(27) Unknown terpene like compound 1	32.84	1674	x		x			
(28) Unknown terpene like compound 2	33.46	1703	x		x			
(29) Unknown tetralin isomer	33.75	1710	x		x			
(30) Unknown aromatic isomer	34.22	1721	x		x			
(31) Unknown compound 5	34.34	1724	x		x			
(32) Unknown di-terpene	34.78	1734	x		x			
(33) Unknown terpene like compound 3	35.31	1746	x		x			
(34) Unknown compound 6	38.73	2101				x		x
(35) Unknown compound 7	42.04	2360	x		x			
**Number of detected compounds (*n*)**			**27**	**15**	**26**	**13**	**16**	**18**

*# = Compound number, Chry = *Chryseobacterium*, Dye = *Dyella*, Jant = *Janthinobacterium*, Tsuk = *Tsukamurella*, MIX Chry + Tsuk = pairwise combination of *Chryseobacterium* + *Tsukamurella*. MIX Jant + Dye = pairwise combination of *Dyella* + *Janthinobacterium*.*

*X = detected.*

*RT^∗^ = Retention time, the RT value stated is the average.*

*ERI^∗∗^ = Experimental retention index value, the RI value stated is the average.*

### Effect of Inter-specific Interactions on Bacterial Volatile Blend Composition

Volatolomic analysis on monocultures and pairwise combinations of *Chryseobacterium* sp. AD48 with *Tsukamurella* sp. AD106 revealed that the volatile composition of the monocultures differed from that of the mixtures (**Figure 1A**; **Table 2**). Clear separations between controls, monocultures and pairwise combinations of *Chryseobacterium* sp. AD48 with *Tsukamurella* sp. AD106 were obtained in PCA score plots (**Figure 1A**). The volatile composition of the pairwise combinations resembled that of the monocultures of *Chryseobacterium* sp. AD48 (**Figure 1A**; **Table 2**). The indole produced by the monoculture of *Chryseobacterium* sp. AD48 was not detected in the interactions (**Table 2**).

The analysis on the volatiles emitted by monocultures and pairwise combinations of *Dyella* sp. AD56 and *Janthinobacterium* sp. AD80 revealed that the volatile profiles of the monocultures differed from that of the mixtures (**Figure 1B**; **Table 2**). Different PCA score plots were obtained between controls, monocultures and pairwise combinations of *Dyella* sp. AD56 with *Janthinobacterium* sp. AD80 (**Figure 1B**). A higher number of volatile compounds were detected in the pairwise combinations of these two bacteria. However, the higher number of detected volatiles is most probably due to the combination of the volatile blends of these two bacterial isolates. We did not detect any novel or different volatile compounds which production was triggered during the pairwise interaction of these two bacteria. Interestingly the volatile compound cyclopentene produced by the monocultures of *Dyella* sp. AD56 and *Janthinobacterium* sp. AD80 was not detected in the interactions (**Table 2**).

### Effect of Bacterial Volatiles on Fungal and Oomycetal Growth

**FIGURE 2:**
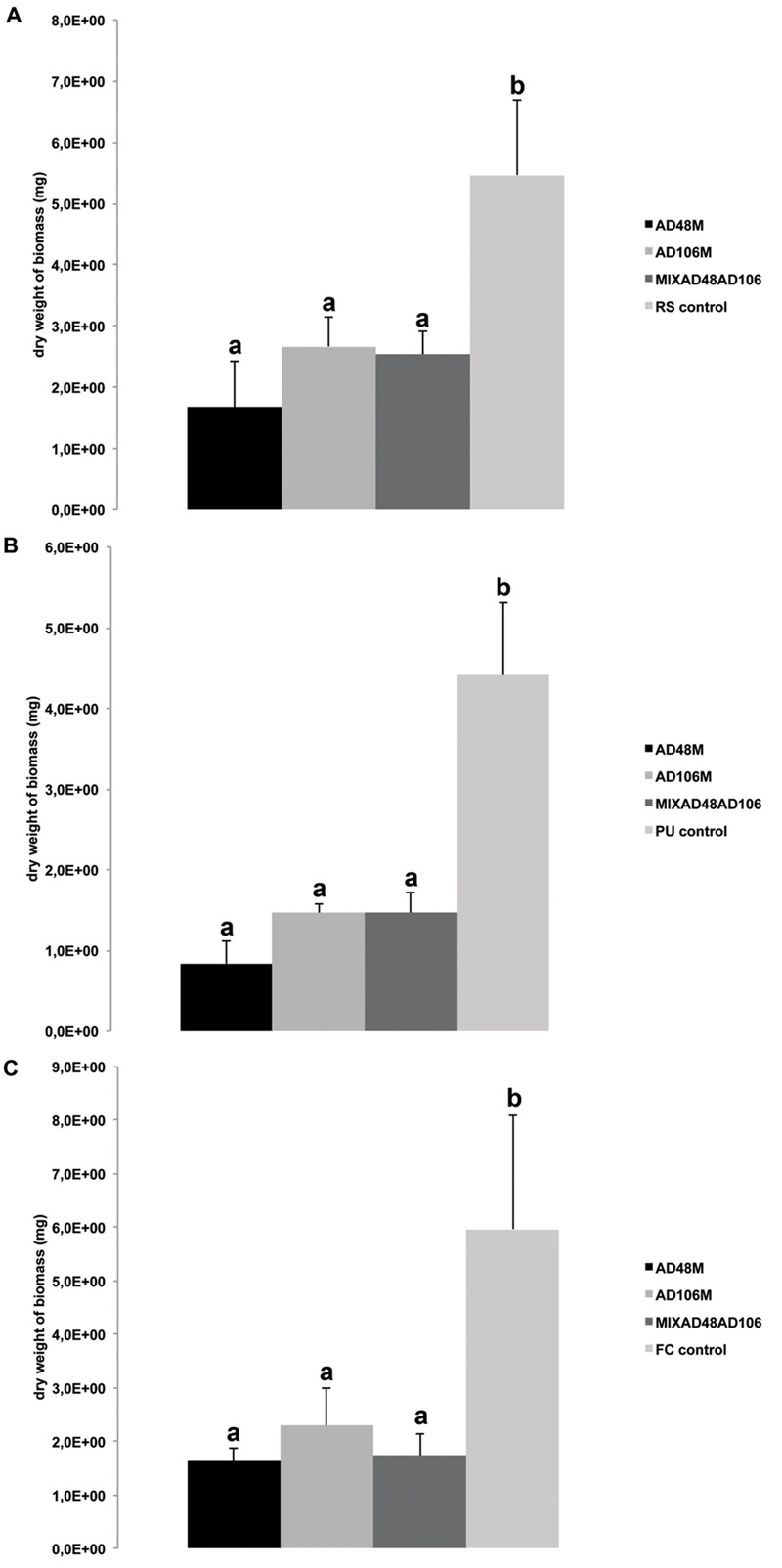
**Effect of volatiles produced by monocultures and mixtures of *Tsukamurella* sp. AD106 and *Chryseobacterium* sp. AD48 on growth of eukaryotic plant-pathogens.** Bars represent the average values for fungal and oomycetal biomass dry weight and error bars represent standard deviation of the mean. **(A)** Dry weight of *Rhizoctonia solani;* **(B)** dry weight of *Pythium ultimum*; **(C)** dry weight of *Fusarium culmorum*. Significant differences between treatments and the control are indicated by different letters (one-way ANOVA, *post hoc* Tukey test *p* < 0.05).

**FIGURE 3:**
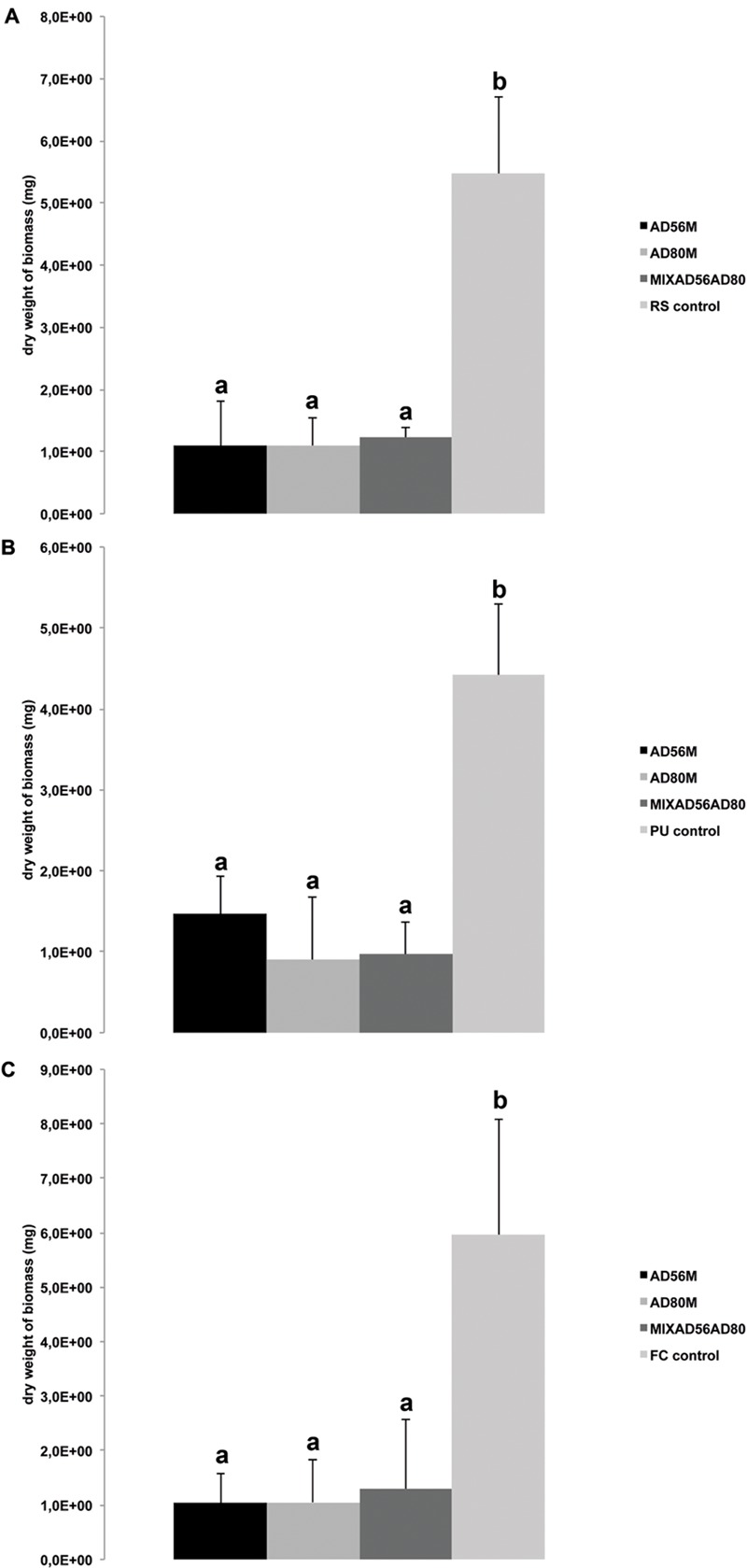
**Effect of volatiles produced by monocultures and mixtures of volatile emitting *Dyella* sp. AD56 and *Janthinobacterium* sp. AD80 on growth of eukaryotic plant-pathogens.** Bars represent the average values for fungal and oomycetal biomass dry weight and error bars represent standard deviation of the mean. **(A)** Dry weight of *R. solani*; **(B)** dry weight of *P. ultimum*; **(C)** dry weight of *F. culmorum.* Significant differences between treatments and the control are indicated by different letters (one-way ANOVA, *post hoc* Tukey test *p* < 0.05).

Volatiles produced by all treatments including monocultures and pairwise combinations of the selected bacteria revealed strong growth inhibition of the plant pathogenic fungi and oomycete. The dry biomass of fungi and oomycete exposed to bacterial volatiles was significantly reduced as compared to the controls without bacterial volatiles (**Table 3**; **Figures 2** and **3**).

**Table 3:** Effect of bacterial volatiles on fungal and oomycetal biomass production (mg/dry weight of fungal/oomycetal biomass).

Treatment	*F. culmorum*	*P. ultimum*	*R. solani*
**Monocultures**			
*Chryseobacterium* sp. AD48	1.63±0.25^∗^	0.83±0.28^∗^	1.67±0.75^∗^
*Dyella* sp. AD56	1.03±0.55^∗^	1.47±0.47^∗^	1.1±0.71^∗^
*Janthinobacterium* sp. AD80	1.05±0.77^∗^	0.9±0.44^∗^	1.1±0.44^∗^
*Tsukamurella* sp. AD106	2.3±0.69^∗^	1.47±0.12^∗^	2.67±0.47^∗^
**Interactions**			
*Chryseobacterium* sp. AD48 + *Tsukamurella* sp. AD106	1.73±0.4^∗^	1.47±0.25^∗^	2.53±0.37^∗^
*Janthinobacterium* sp. AD80 + *Dyella* sp. AD56	1.3±1.27^∗^	0.97±0.40^∗^	1.23±0.15^∗^
**Controls**	5.97±2.13	4.42±0.88	5.47±1.23

*Data represent mean and standard deviation of three replicates.*

*Asterisk indicates significant differences between the treatments and the respective control (one-way ANOVA, *post hoc* Tukey test *p* < 0.05).*

### Effect of Bacterial Volatiles on the Growth and Behavior of Target Bacteria

**FIGURE 4:**
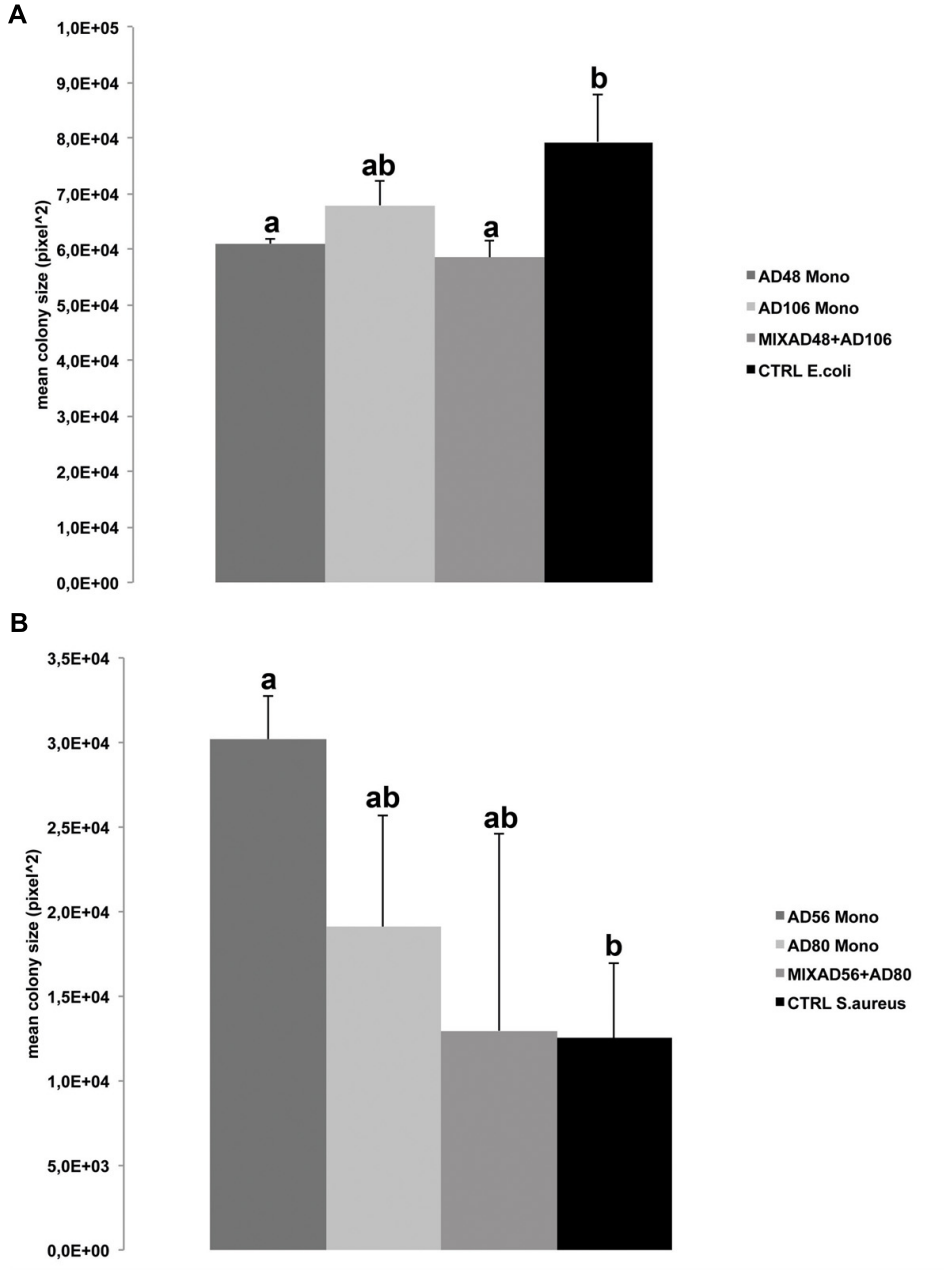
**Effect of volatiles produced by monocultures and pair-wise combinations of the four selected rhizosphere bacterial strains on average colony size of the target bacteria. (A)** Mean colony size of *Escherichia coli* WA321 exposed to volatile compounds of *Chryseobacterium* sp. AD48 and *Tsukamurella* sp. AD106 and the mixture of both bacteria. **(B)** Mean colony sizes of *Staphylococcus aureus* 533R4 exposed to volatile compounds of *Dyella* sp. AD56, *Janthinobacterium* sp. AD80 and the mixture of both bacteria. Significant differences between treatments and the control are indicated by different letters (one-way ANOVA, *post hoc* Tukey test *p* < 0.05). Data represented are the mean of three replicates, error bars represent standard deviation of the mean.

Volatiles emitted by *Chryseobacterium* sp. AD48 and the mixture of *Chryseobacterium* sp. AD48 and *Tsukamurella* sp. AD106 inhibited the growth of *E. coli* WA321 significantly as compared to the control (**Figure 4A**). This observation is in agreement with the observed volatolomic profile (**Figure 1A**) which revealed that the volatolomic profile of the mixture is dominated by the volatiles produced by the monoculture of *Chryseobacterium* sp. AD48.

Besides growth inhibition we observed significant growth promotion of *S. aureus* 533R4 when exposed to volatiles emitted by the monocultures of *Dyella* sp. AD56 (**Figure 4B**).

Changes in colony morphology of *S. marcescens* P87 were observed when exposed to volatiles emitted by *Chryseobacterium* sp. AD48 and to volatiles emitted by the mixtures of *Dyella* sp. AD56 with *Janthinobacterium* sp. AD80. The *S. marcescens* P87 colonies were more circular and round shaped (Supplementary Figure S8). However, no significant effects of bacterial volatiles on the growth of the target bacteria were also observed (Supplementary Figure S9).

### Effect of Pure Individual Volatile Compounds on the Growth and Colony Morphology of Target Bacteria

**FIGURE 5:**
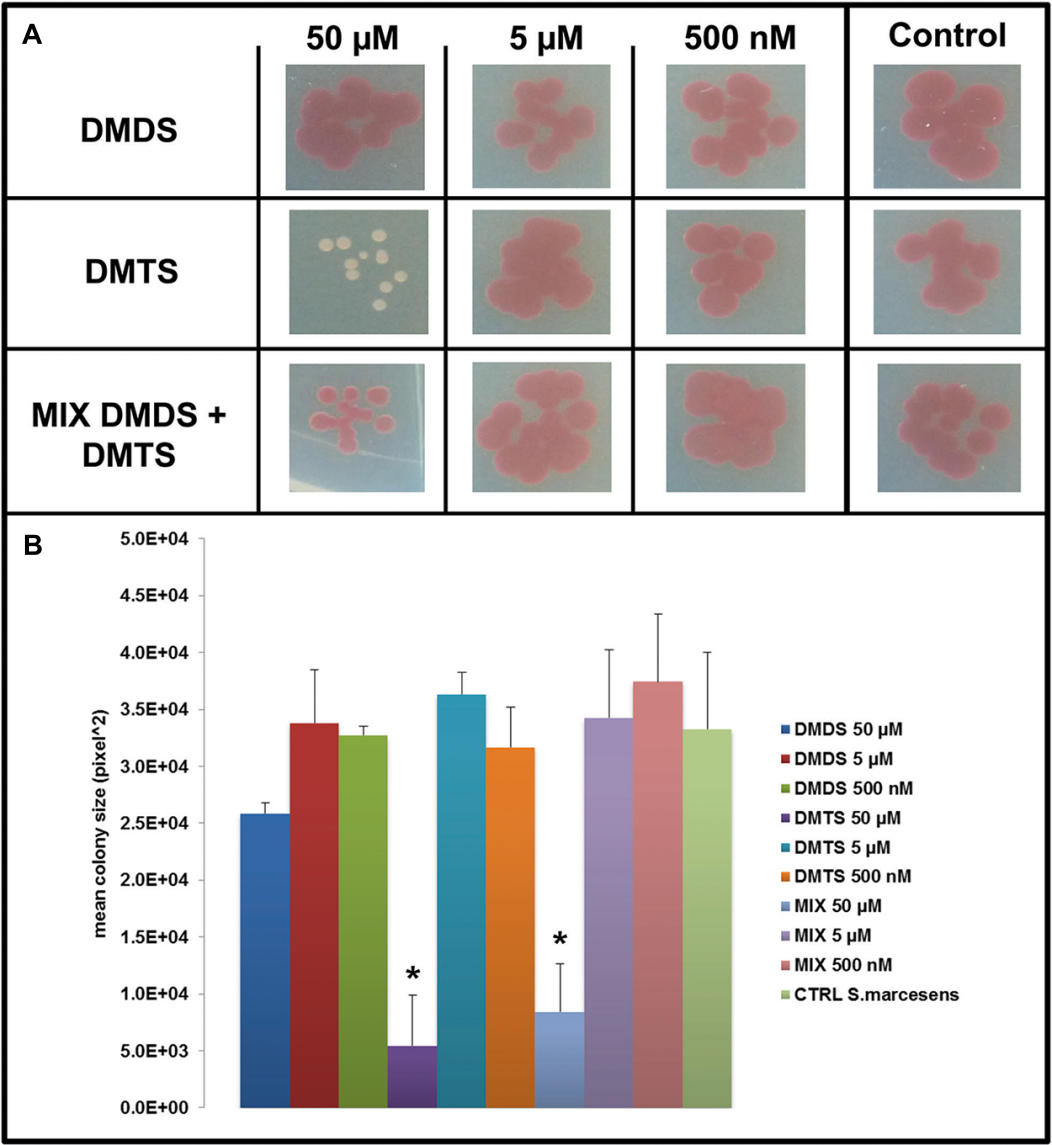
**Effect of dimethyl disulfide (DMDS), dimethyl trisulfide (DMTS), and the mixture of both volatile compounds (DMDS + DMTS) on colony development of *S. marcescens.* (A)** Colony morphology and growth of *S. marcescens* P87 after 4 days of incubation. The pure volatile compounds were applied in a concentration ranging from 500 nM to 50 μM. Control *S. marcescens* P87 grown without exposure to the compounds. **(B)** Mean colony sizes of *S. marcescens* P87 exposed to volatile compounds of DMDS, DMTS, and the mixture of both volatile compounds (DMDS + DMTS). Asterisk indicates significant differences between the treatments and the control (one-way ANOVA, *post hoc* Tukey test *p* < 0.05). Data represented are the mean of three replicates, error bars represent standard deviation of the mean.

**FIGURE 6:**
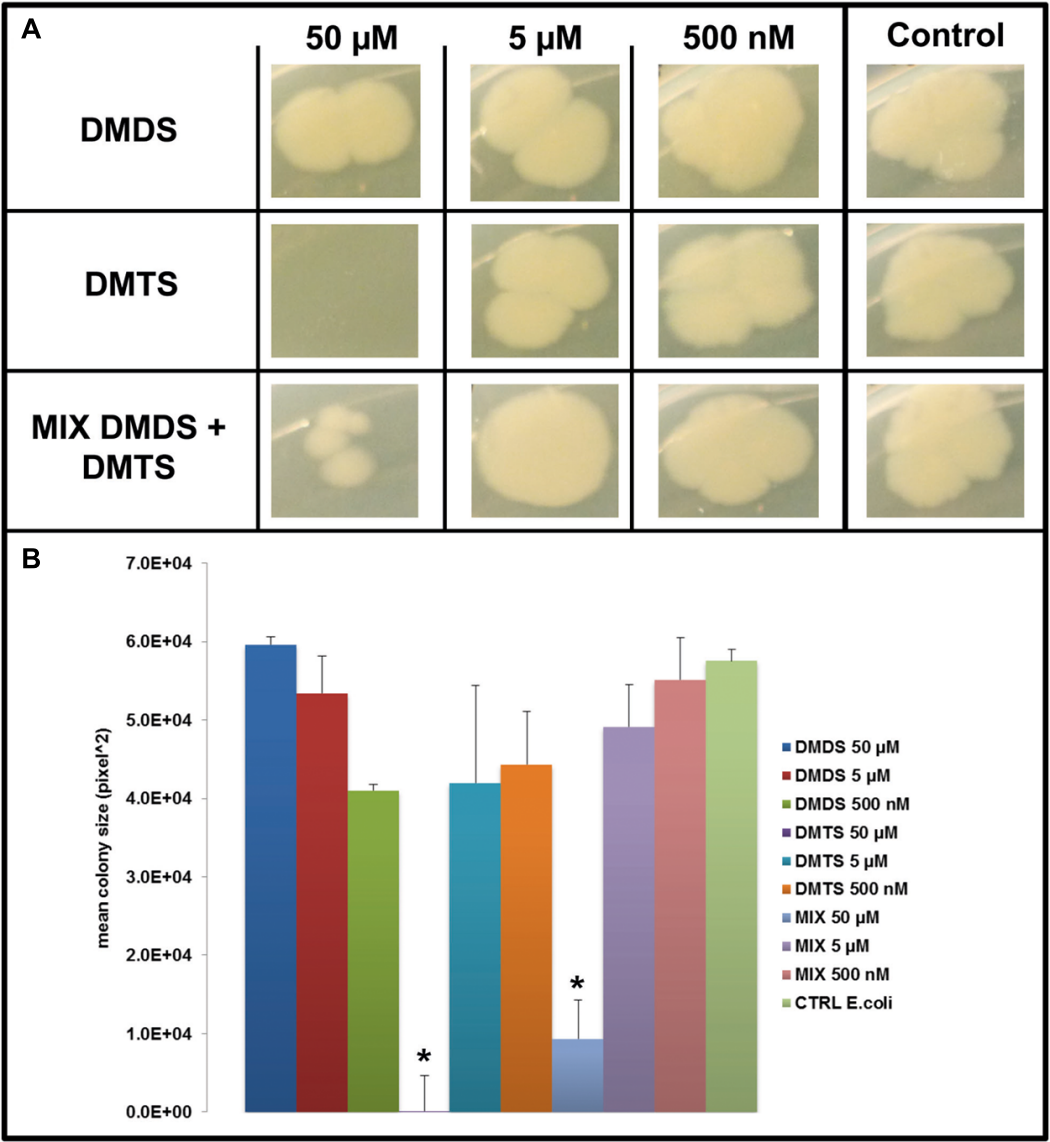
**Effect of DMDS, DMTS, and the mixture of both volatile compounds (DMDS + DMTS). (A)** Colony morphology and growth of *E. coli* WA321 after 4 days of incubation. The pure volatile compounds were applied in a concentration ranging from 500 nM to 50 μM. Control *E. coli* WA321 grown without exposure to the compounds. **(B)** Mean colony sizes of *E. coli* WA321 exposed to volatile compounds of DMDS, DMTS, and the mixture of both volatile compounds (DMDS + DMTS). Asterisk indicates significant differences between the treatments and the control (one-way ANOVA, *post hoc* Tukey test *p* < 0.05). Data represented are the mean of three replicates, error bars represent standard deviation of the mean.

**FIGURE 7:**
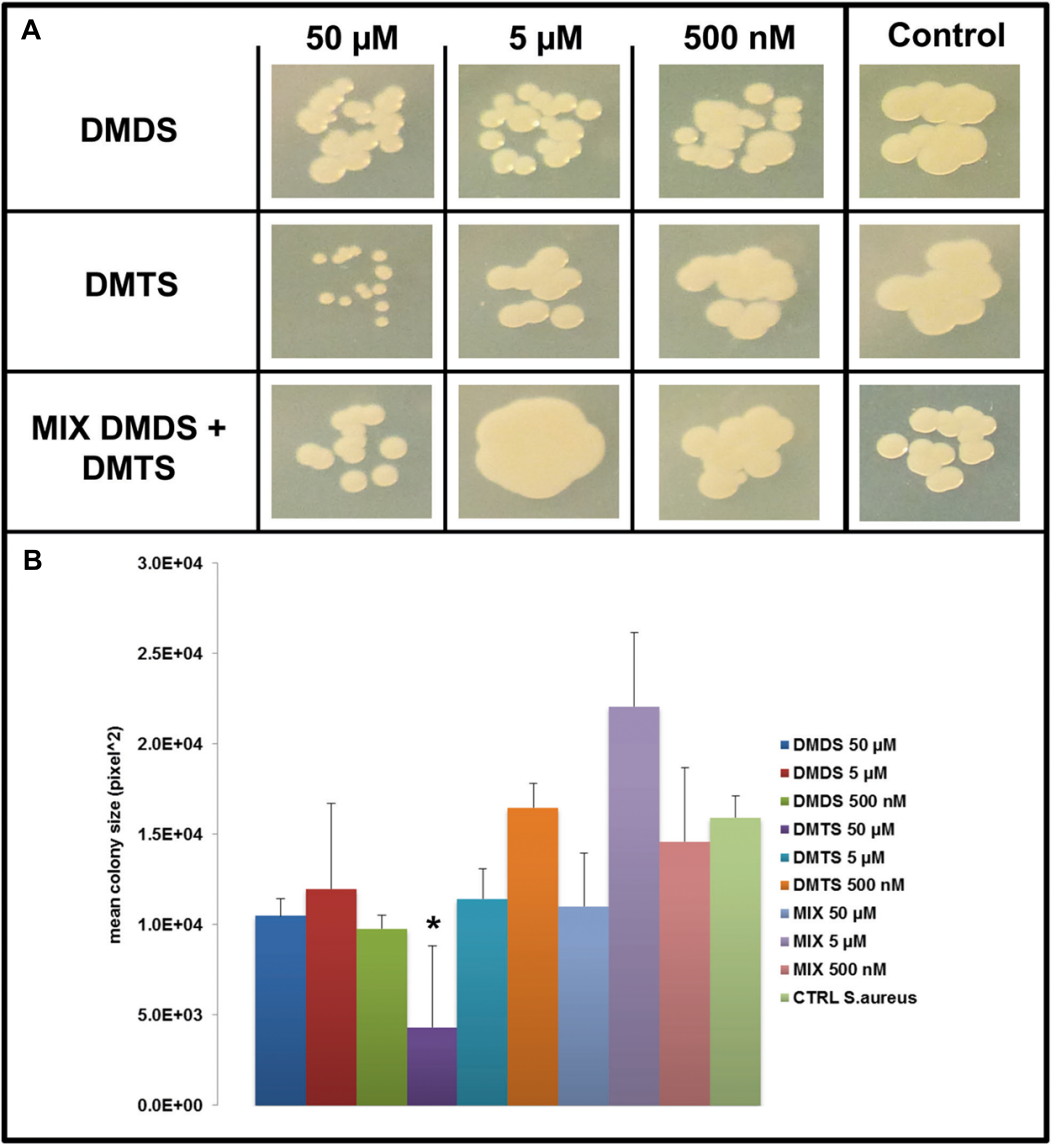
**Effect of DMDS, DMTS, and the mixture of both volatile compounds (DMDS + DMTS). (A)** Colony morphology and growth of *S. aureus* 533R4 after 4 days of incubation. The pure volatile compounds were applied in a concentration ranging from 500 nM to 50 μM. Control *S. aureus* 533R4 grown without exposure to the compounds. **(B)** Mean colony sizes of *S. aureus* 533R4 exposed to volatile compounds of DMDS, DMTS, and the mixture of both volatile compounds (DMDS + DMTS). Asterisk indicates significant differences between the treatments and the control (one-way ANOVA, *post hoc* Tukey test *p* < 0.05). Data represented are the mean of three replicates, error bars represent standard deviation of the mean.

We applied a two-compartment Petri dish testing system (Supplementary Figure S4) in which the model organisms could grow without direct physical contacts to the tested pure volatile compounds. After 4 days of growth *S. marcescens* P87 colonies were small and showed a white phenotype when exposed to 50 μM of DMTS, indicating the lack of prodigiosin production (**Figure 5A**). Furthermore we observed significant inhibition of growth of *S. marcescens* P87, *E. coli* WA321 and *S. aureus* 533R4 when exposed to 50 μM of DMTS (**Figures 5**–**7**).

Exposure to DMDS did not reveal any significant growth inhibiting or changes in colony morphology at all concentrations tested (500 nM, 5 and 50 μM). The mixture of DMDS and DMTS resulted in growth inhibition of *S. marcescens* P87 and *E. coli* WA321 at 50 μM concentration. However, the pigmentation in *S. marcescens* P87 was not affected by the mixture of these compounds. The two lowest applied concentrations 5 and 0.5 μM of DMTS and DMDS and the mixture of both compounds did not reveal any effect on colony morphology or growth of the tested bacteria (**Figures 5**–**7**).

## Discussion

Bacteria coexist with many different species in a heterogeneous and challenging soil environment (Gans et al., 2005). In this environment inter-specific interactions between microorganisms are ongoing and are a key factor for their spatial distribution (Keller and Surette, 2006). To cope with the competitive conditions, bacteria developed different survival strategies such as the production of secondary metabolites with inhibitory capacity (Hibbing et al., 2010; Cornforth and Foster, 2013). Most of the studies on bacterial secondary metabolites so far were focused on non-volatile compounds (Korpi et al., 1998; Foster and Bell, 2012). However, bacteria do also release complex blends of VOCs. Yet, the effect of inter-specific interactions on volatiles production and composition is still unknown (Garbeva et al., 2014a).

Here, we compared the volatile blends emitted by four phylogenetically different soil-bacteria either grown in monocultures or in pairwise combinations. Our results revealed that the blend of volatiles emitted during pairwise combinations differed from the volatile blends of the respective monocultures. Yet, the volatile blend of the mixtures mostly included volatiles compounds produced by monocultures, although some compounds produced by the monocultures were not detected in mixtures. For example dimethyl sulfoxide produced by *Tsukamurella* sp. AD106 was not detected in the mixture with *Chryseobacterium* sp. AD48. Another interesting example is indole which was produced by the monocultures of *Chryseobacterium* sp. AD48 but was not detected in the presence of *Tsukamurella* sp. AD106. Indole is a very well-studied compound and has been reported to be produced by about 85 different bacterial species including *Chryseobacterium* sp. (Yamaguchi and Yokoe, 2000; Lee and Lee, 2010). Indole and its derivatives [quinolones and (*S*)-3-hydroxytridecan-4-one] are involved in intercellular and multispecies signaling controlling diverse bacterial physiological properties like sporulation, plasmid stability, biofilm formation, drug resistance and virulence (Wang et al., 2001; Di Martino et al., 2003; Diggle et al., 2006; Nikaido et al., 2008; Lee et al., 2009; Lee and Lee, 2010). In addition, indole has been shown to have inhibitory activities on fungal growth (*Aspergillus niger*) and plant growth stimulating properties (*Arabidopsis thaliana*; Kamath and Vaidyanathan, 1990; Blom et al., 2011). In general indole is known to be a stable compound in the producing bacteria, however, many non-indole producing bacteria are able to modify and to degrade indole (Shimada et al., 2013; Lee et al., 2015). The fact that indole was not detected during the interaction of *Chryseobacterium* sp. AD48 with *Tsukamurella* sp. AD106 suggests that the production of such signaling compounds in nature depends strongly on the inter-specific interactions. Similar result was observed for the compound cyclopentene produced by the monocultures of *Dyella* sp. AD56 and *Janthinobacterium* sp. AD80 but not produced during the interaction of these two bacteria. With the volatolomic methods applied in this study we were able to detect 35 compounds from which 19 were tentatively identified. This discrepancy between numbers of detected and identified compounds shows that the identification of bacterial volatiles is yet a challenging and time demanding task, even with the use of sophisticated programs and software for metabolomics data analysis. Hence, the produced volatile blends are very complex and consist of a mixture of many unknown and difficult to identify compounds (Tait et al., 2014). Most of the VOCs that were tentatively identified within this study (∼58%) contained sulfur (e.g., methyl thiocyanate, DMDS, DMTS, dimethyl tetrasulfide, etc.). The high abundance of sulfur containing volatiles in this study can be related to the cultivation of the tested bacteria on 1/10th TSBA growth media. Several studies indicated that the composition of the volatile blend greatly depends on the growth media composition and the growth conditions (Schulz et al., 2004; Schulz and Dickschat, 2007; Blom et al., 2011; Garbeva et al., 2014b). The high amount of dimethyl di- and trisulfide detected in both monocultures and interactions indicate that these compounds are commonly produced. Many studies have shown that bacterial volatiles play a major role in soil fungistasis (Zou et al., 2007; Garbeva et al., 2011a, 2014b; van Agtmaal et al., 2015). Indeed our results revealed that the fungal and oomycete tested organism are sensitive to bacterial volatiles and were inhibited significantly by all monocultures and pairwise combinations. The observed fungal and oomycetal growth inhibition is most probably related to sulfur containing volatiles. Sulfur containing volatiles like dimethyl di- and trisulfide have been shown to effect fungi and are able to inhibit the growth of different plant pathogenic fungi (Kai et al., 2009; Li et al., 2010; Huang et al., 2012; Wang et al., 2013; Garbeva et al., 2014b; Kanchiswamy et al., 2015).

While many study tested the effect of bacterial volatiles on various fungi, little is known so far on the effect of bacterial volatiles on other bacteria. In this study *E. coli* WA321 was inhibited by the volatiles emitted by *Chryseobacterium* sp. AD48 and the mixture of *Chryseobacterium* sp. AD48 with *Tsukamurella* sp. AD106. The observed growth promotion of *S. aureus* 533R4 was caused by the volatiles emitted by *Dyella* sp. AD56. However, this growth promotion was not observed by the volatiles emitted during the interaction of *Dyella* sp. AD56 with *Janthinobacterium* sp. AD80 correlating with a shift in volatile blend composition. Interestingly volatiles emitted by the monocultures of *Chryseobacterium* sp. AD48 and the mixture of *Dyella* sp. AD56 with *Janthinobacterium* sp. AD80 induced changes in colony morphology of *S. marcescens* P87. Our previous high-throughput screening for production of non-volatile antimicrobial compounds revealed that all four bacteria used here, showed induced antibacterial activity during pairwise interactions as compared to monocultures (Tyc et al., 2014). This was not observed in the present study, as we didn’t observed novel produced volatile compounds during the pairwise interactions. Therefore, it’s questionable if volatiles solely play an important role as a competitive strategy between bacteria. However, it is possible that volatiles have synergistic or additive effect to other non-volatile antibacterial compounds (Schmidt et al., 2015). Many bacteria are known to emit inorganic volatiles like CO_2_, NH_3_, HCN, which also have biological activities and can have an additive effect (Effmert et al., 2012). However, such compounds were not detected in this study as significant volatile compounds.

Here, we tested two commonly produced bacterial volatile compounds for their effect on the target bacteria. The experiments with pure DMTS revealed strong growth inhibition on all tested bacterial model organisms, when applied in a concentration of 50 μM. Bacterial growth suppression was already reported for DMDS emitted by *Pseudomonas* strains against the crown-gall diseases causing *Agrobacterium* sp. (Dandurishvili et al., 2011; Popova et al., 2014). Dimethyl trisulfide effected colony morphology and pigmentation in *S. marcescens* P87 when applied in a concentration of 50 μM. Volatiles exposed colonies showed reduced growth and white coloration indicating the lack of prodigiosin production. It is plausible that this observation is related to the inhibition of quorum-sensing as previously reported by Morohoshi et al. (2007), Chernin et al. (2011). However, the effective concentration of 50 μM DMTS is most probably very high and far away from the concentrations in which those volatile compounds are produced in nature (Groenhagen et al., 2013) as we did not observed this effect in the experiments where *S. marcescens* P87 was exposed to the volatile blend produced by bacteria. The biological relevant concentration of volatile compounds remains to be determined in future studies.

## Conclusion

This work revealed that inter-specific bacterial interactions affect volatile blend composition. This observed change is most probably related to the combination of volatile compounds produced by each isolate rather than triggering the production of novel volatiles as the volatile blend was composed of the mixture of the respective interacting bacteria. Furthermore, the loss of production of certain compounds during pairwise interaction suggests that the production of volatile signaling compounds (e.g. indole) in nature is influenced by inter-specific interactions. While fungi and oomycetes showed to be very sensitive to bacterial volatiles the effect of volatiles on bacteria varied greatly between no effects, growth inhibition to growth promotion depending on the volatile blend composition.

## Conflict of Interest Statement

The authors declare that the research was conducted in the absence of any commercial or financial relationships that could be construed as a potential conflict of interest.

## Footnotes

1 http://bioinformatics.charite.de/mvoc/

2 http://www.nist.gov
