# Supplementary material for: Volatiles in Inter-Specific Bacterial Interactions
Source: Front Microbiol. 2015 Dec 18;6:1412. doi: 10.3389/fmicb.2015.01412 (PMC4683202; doi:10.3389/fmicb.2015.01412)
Supplement: Supplementary file 1 [file Data_Sheet_1.PDF]

Supplementary Material

Volatiles in inter-specific bacterial interactions

Olaf Tyc[1,2]*, Hans Zweers[1], Wietse de Boer[1,2] and Paolina Garbeva[1]

[1] Department of Microbial Ecology, Netherlands Institute of Ecology (NIOO-KNAW), PO Box 50, 6700 AB, Wageningen, Netherlands

[2] Department of Soil Quality, Wageningen University & Research Centre (WUR), PO Box 47, 6700 AA, Wageningen, Netherlands

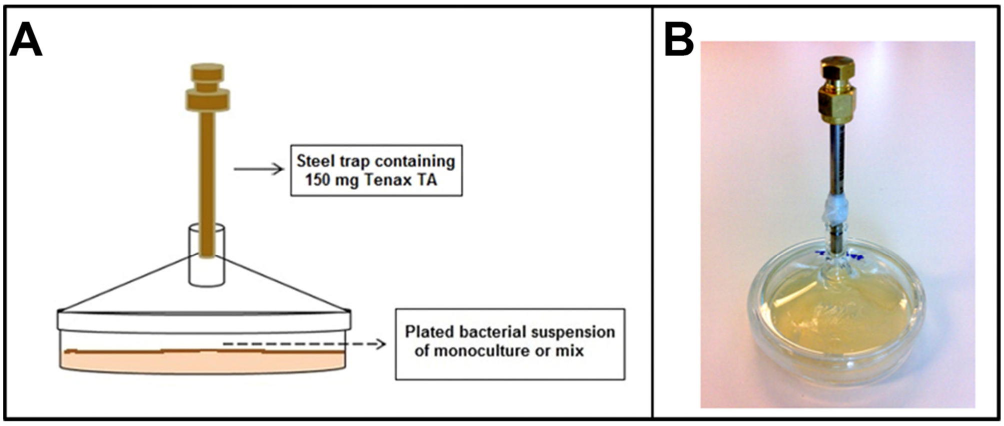

Figure S1: Used glass-petri dish system for bacterial volatile organic compounds (VOCs) trapping. **A:** illustration of the glass-petri dish; **B:** photograph of a inoculated glass-petri dish with a connected Tenax TA steel trap for volatile organic compound trapping.

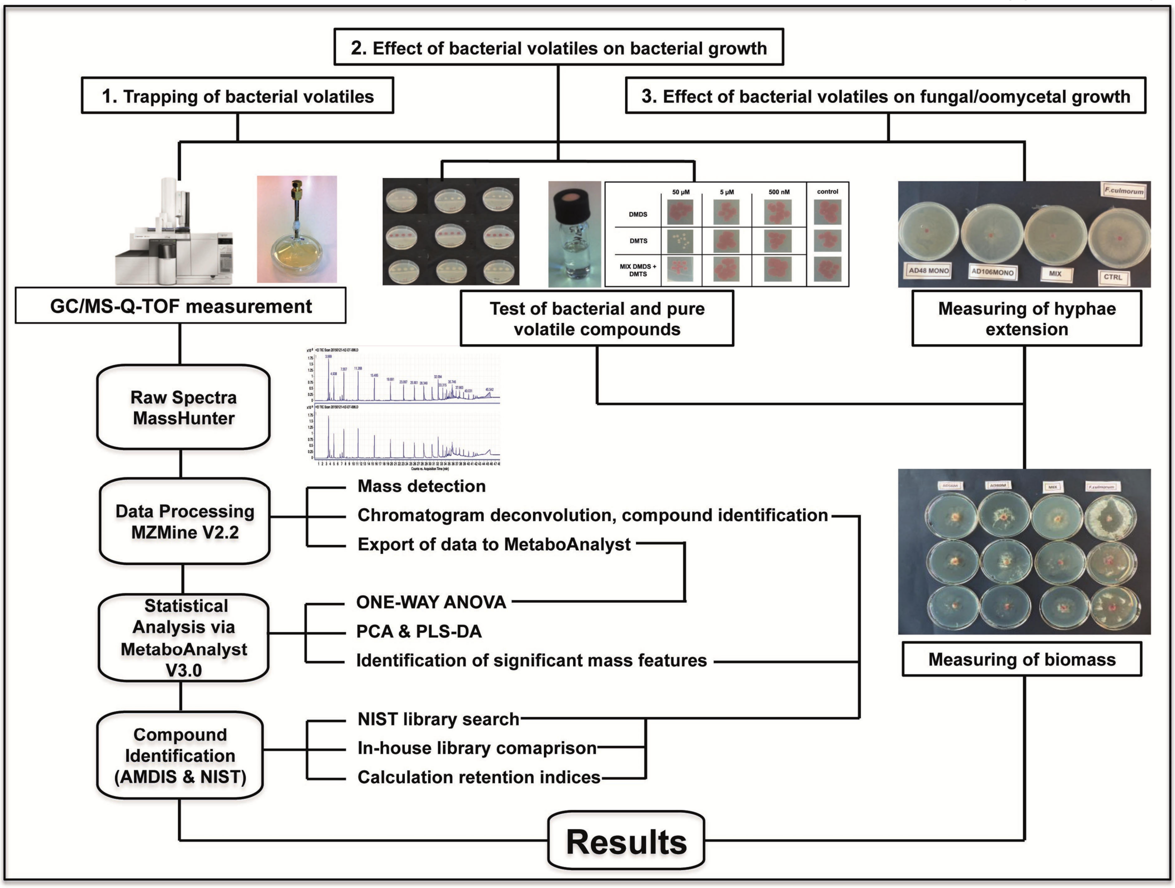

Figure S2: Workflow of the volatolomics analysis performed in this study. In total three experimental parts were carried out: 1. Volatile trapping and GC/MS-Q- TOF combined with statistical analysis, 2. Effect of bacterial volatiles on bacterial growth and colony morphology and 3. Effect of bacterial volatiles on fungal and oomycetal model organisms.

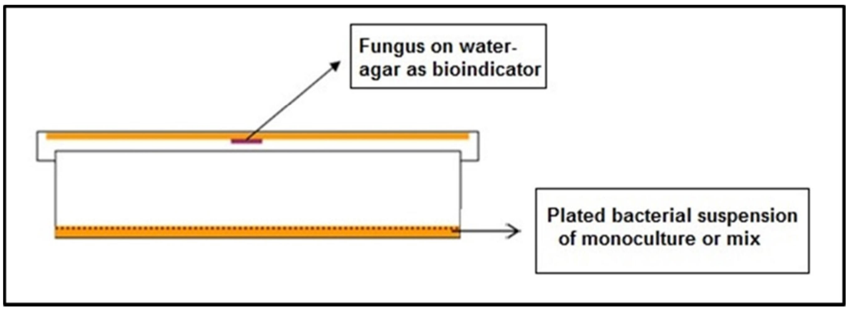

Figure S3: Used top-bottom-petri dish system for experiments to elucidate fungal inhibitory capacities of the produced bacterial volatiles.

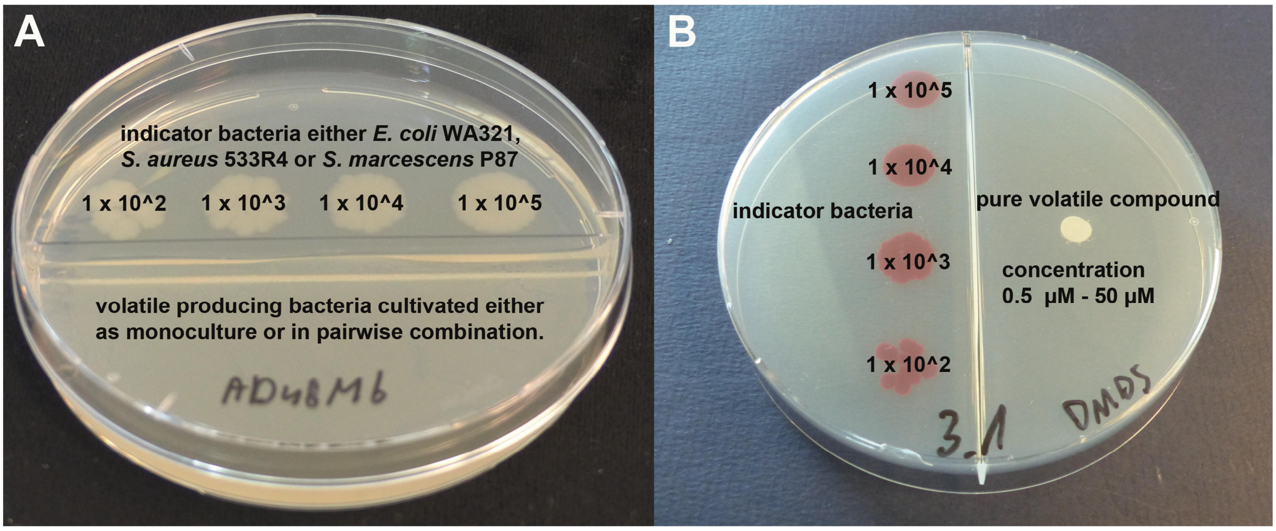

Figure S4: Two-compartment petri dish system used in bacterial volatile and pure volatile compound experiments. **A:** to elucidate the effect of bacterial volatiles on growth and colony morphology of *E. coli* WA321, *S. aureus* 533R4 and *S. marcescens* P87 **B:** to elucidate the effect of pure volatile compounds dimethyl di- and tri- sulfide on growth and colony morphology on *E. coli* WA321, *S. aureus* 533R4 and *S. marcescens* P87.

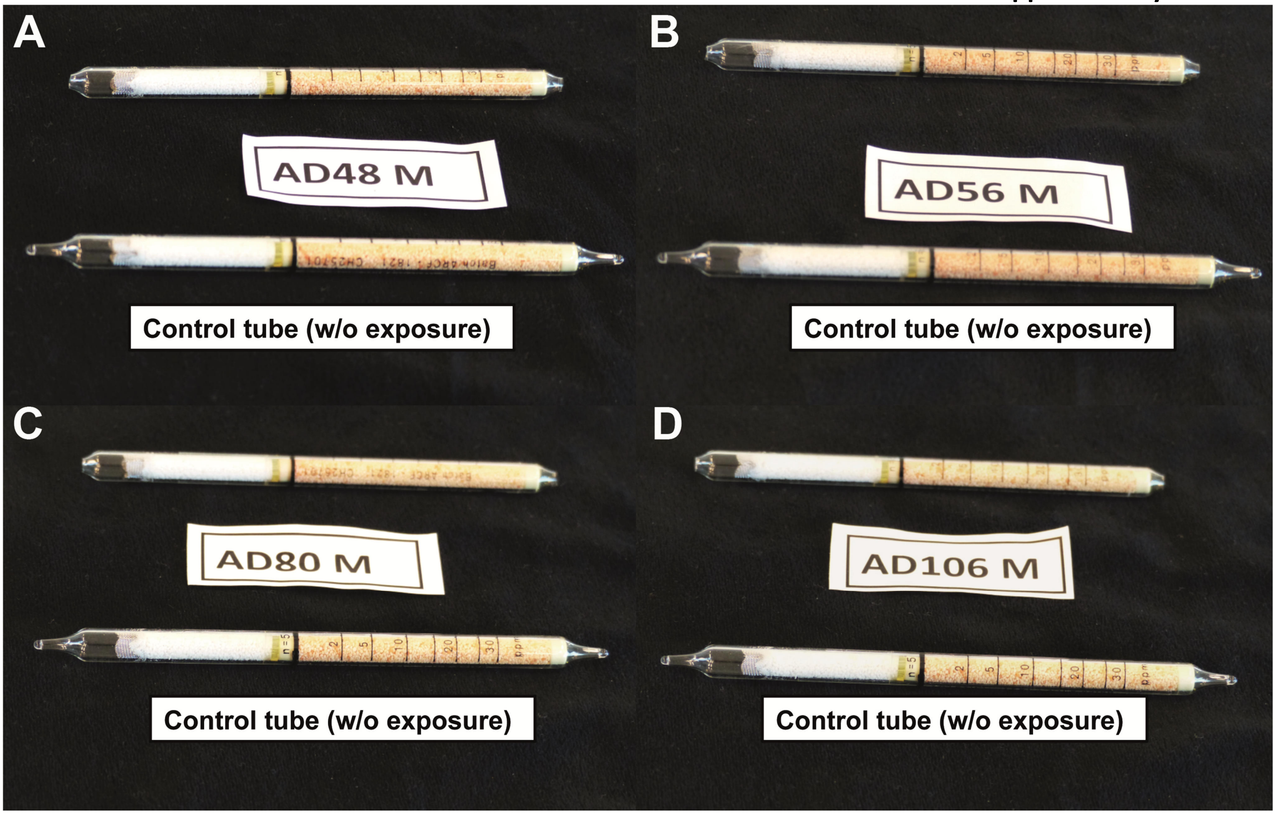

Figure S5: Hydrocyanic-acid tests using Dräger Hydrocyanic acid test tubes in combination with the Dräger accuro® gas detection pump. All bacterial strains were tested negative for the production of Hydrocyanic acid (< 2 ppm). Test result for: **A:** *Chryseobacterium* sp. AD48. **B:** *Dyella* sp. AD56 **C:** *Janthinobacterium* sp. AD80. **D:** *Tsukamurella* sp. AD106.

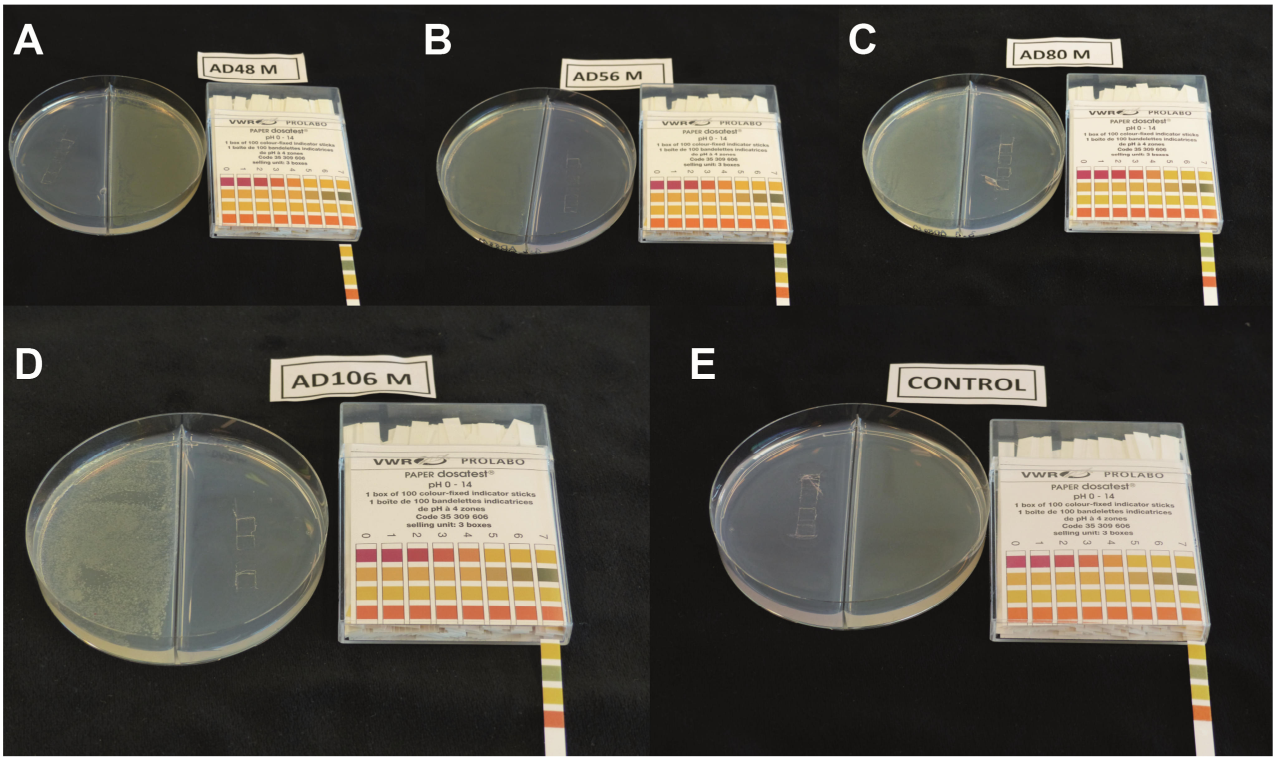

Figure S6: pH- tests using VWR PROLABO dosatest® pH- test strips. All bacterial strains did not change the pH- value of the growth medium where the target organism were grown (pH = 7). Test result for: **A:** *Chryseobacterium* sp. AD48. **B:** *Dyella* sp. AD56 **C:** *Janthinobacterium* sp. AD80. **D:** *Tsukamurella* sp. AD106. **E:** The control, two compartment petri dish without exposure to bacterial volatiles.

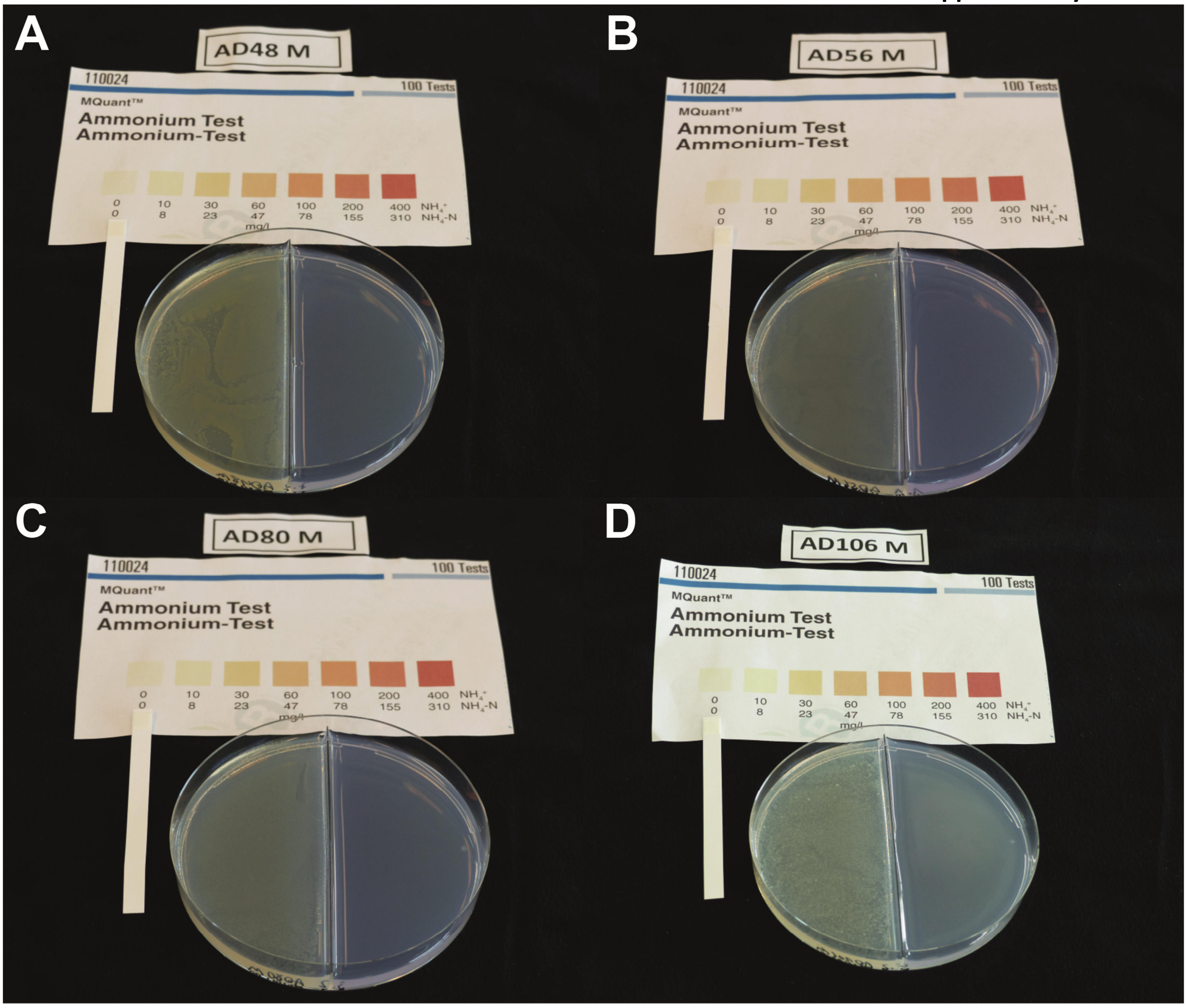

Figure S7: Measurements of ammonia emission using the MQuant™ ammonium test kit. All bacterial strains were tested negative for the production of ammonium. Test result for: **A:** *Chryseobacterium* sp. AD48. **B:** *Dyella* sp. AD56 **C:** *Janthinobacterium* sp. AD80. **D:** *Tsukamurella* sp. AD106.

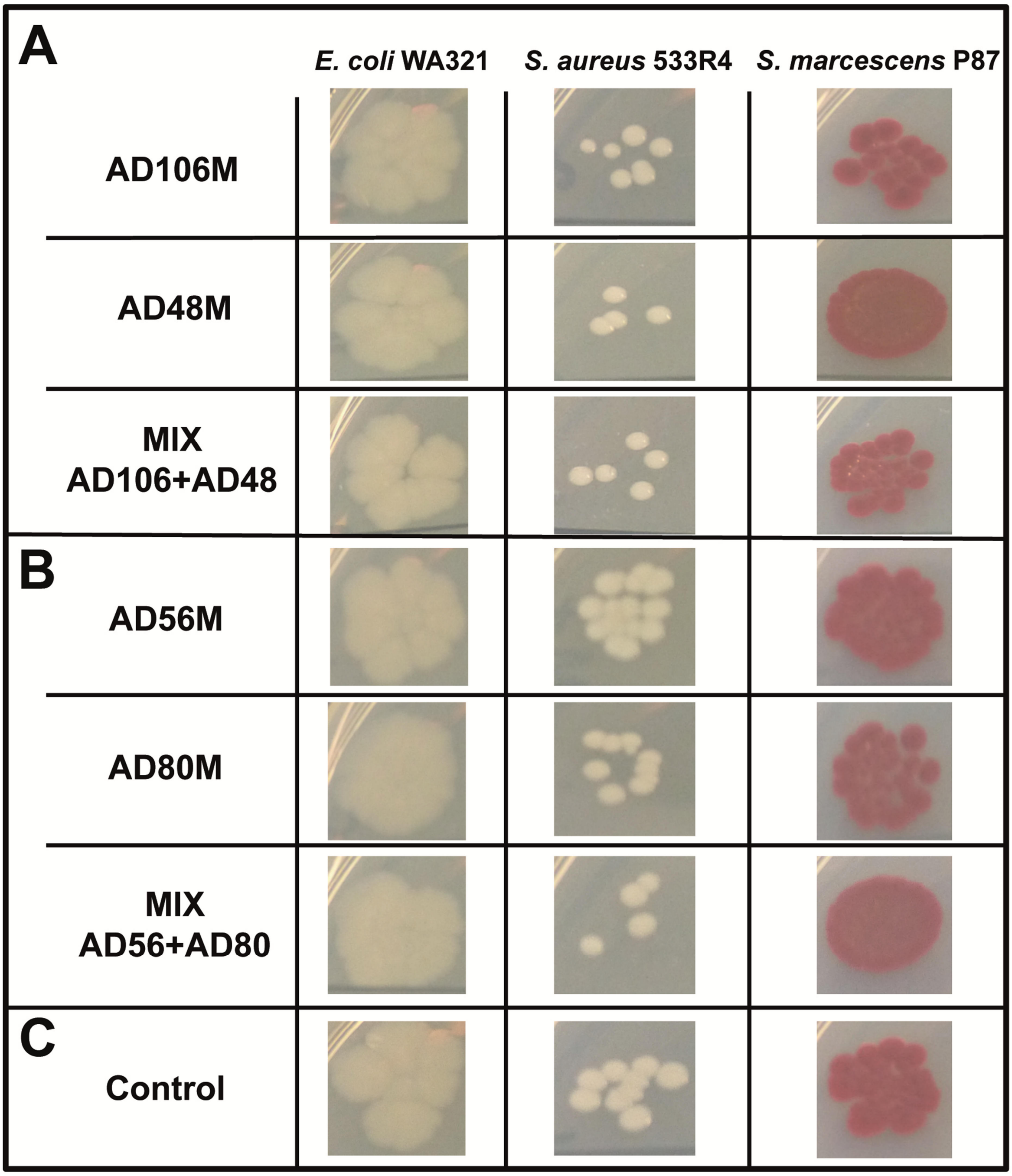

Figure S8: Effect of bacterial volatile compounds on colony morphology on the three model organisms: *E.coli* WA321, *S.aureus* 533R4 and *S. marcescens* P87. **A:** Effect of the produced volatile blend of *Tsukamurella* sp. AD106, *Chryseobacterium* sp. AD48 and the interaction of both bacteria **B:** Effect of the produced volatile blend of *Dyella* sp. AD56, *Janthinobacterium* sp. AD80 and the interaction of both bacteria. **C:** Control without exposure to bacterial volatiles.

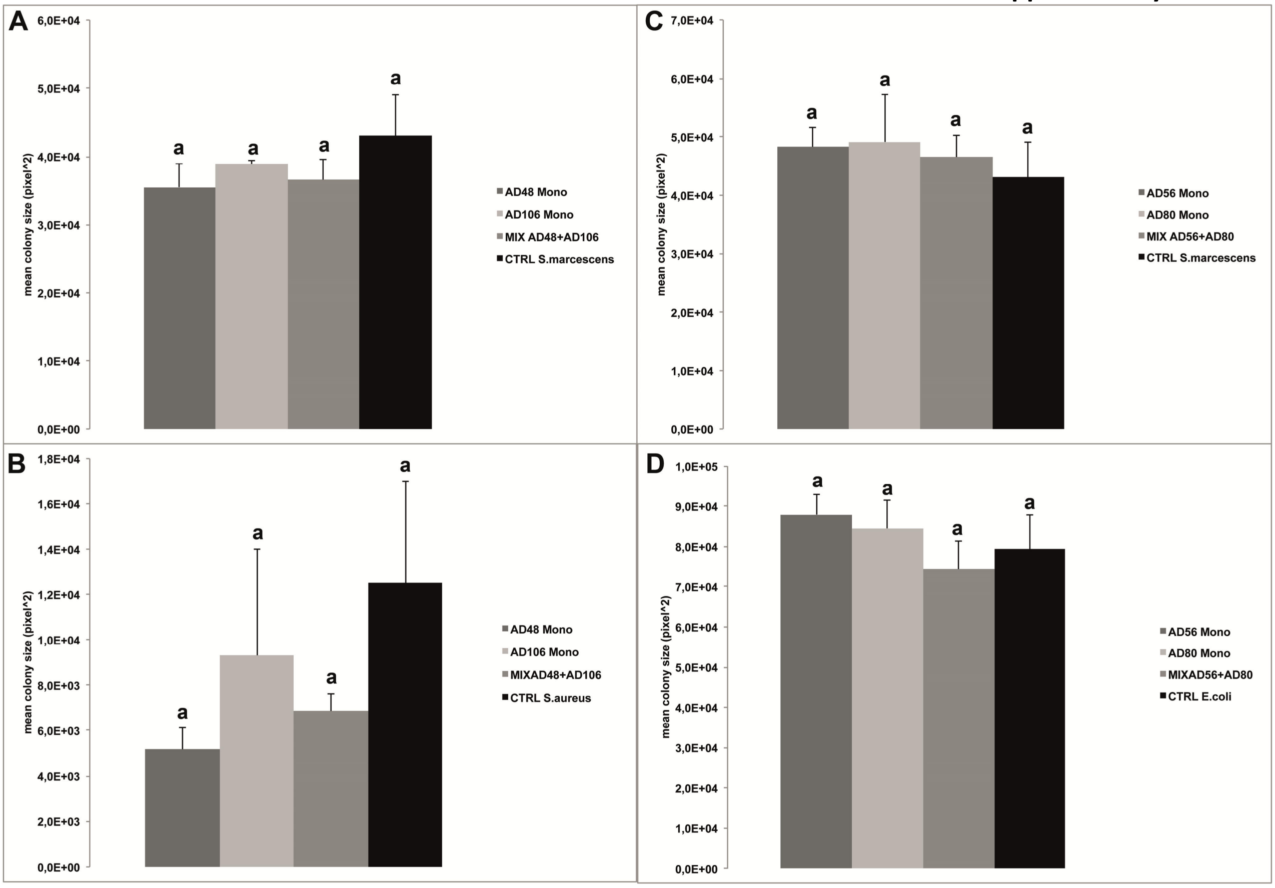

Figure S9: Average colony size of the target organisms. **A:** Mean colony size of *S. marcescens* P87 exposed to volatile compounds of *Chryseobacterium* sp. AD48 and *Tsukamurella* sp. AD106 and the interaction of both bacteria. **B:** Mean colony size of *S. aureus* 533R4 exposed to volatile compounds of *Chryseobacterium* sp. AD48 and *Tsukamurella* sp. AD106 and the interaction of both bacteria. **C:** Mean colony size of *S. marcescens* P87 exposed to volatile compounds of *Dyella* sp. AD56, *Janthinobacterium* sp. AD80 and the interaction of both bacteria. **D:** Mean colony size of *E. coli* WA321 exposed to volatile compounds of *Dyella* sp. AD56, *Janthinobacterium* sp. AD80 and the interaction of both bacteria. Data represented are the mean of three replicates, error bars represent standard deviation (SD).
